# Effect of Side-Specific Valvular Shear Stress on the Content of Extracellular Matrix in Aortic Valves

**DOI:** 10.1007/s13239-016-0280-z

**Published:** 2016-10-05

**Authors:** Napachanok Mongkoldhumrongkul, Najma Latif, Magdi H. Yacoub, Adrian H. Chester

**Affiliations:** Imperial College, NHLI, Heart Science Centre, Harefield, Middlesex, UB9 6JH UK

**Keywords:** Side-specific valve endothelial cells, Aortic side, Ventricular side, Aortic flow (oscillatory flow), Ventricular flow (laminar flow)

## Abstract

Responses of valve endothelial cells (VECs) to shear stresses are important for the regulation of valve durability. However, the effect of flow patterns subjected to VECs on the opposite surfaces of the valves on the production of extracellular matrix (ECM) has not yet been investigated. This study aims to investigate the response of side-specific flow patterns, in terms of ECM synthesis and/or degradation in porcine aortic valves. Aortic and ventricular sides of aortic valve leaflets were exposed to oscillatory and laminar flow generated by a Cone-and-Plate machine for 48 h. The amount of collagen, GAGs and elastin was quantified and compared to samples collected from the same leaflets without exposing to flow. The results demonstrated that flow is important to maintain the amount of GAGs and elastin in the valve, as compared to the effect of static conditions. Particularly, the laminar waveform plays a crucial role on the modulation of elastin in side-independent manner. Furthermore, the ability of oscillatory flow on the aortic surface to increase the amount of collagen and GAGs cannot be replicated by exposure of an identical flow pattern on the ventricular side of the valve. Side-specific responses to the particular patterns of flow are important to the regulation of ECM components. Such understanding is imperative to the creation of tissue-engineered heart valves that must be created from the “appropriate” cells that can replicate the functions of the native VECs to regulate the different constituents of ECM.

## Introduction

Heart valve endothelial cells (VECs) play an important role in maintaining valve integrity, in a similar way as vascular endothelial cells (ECs) do in blood vessels. For instance, VECs regulate inflammatory reactions between blood and the valve,^12^ deliver nutrients to the underlying valve interstitial cells (VICs) and regulate the phenotype of the VICs.^3^, ^25^ Moreover, they release vasodilator and vasoconstrictor agents that affect the contractility of the valve and regulate valve’s mechanical properties.^5^, ^10^, ^15^, ^19^ In addition to the regulation of valvular homeostasis, VECs are also thought to be VIC progenitor cells and replenish VICs *via* the activation of endothelial to mesenchymal transformation (EndMT).^13^, ^14^, ^27^

Although VECs share some function with vascular ECs, studies at the genetic and molecular level reveal different transcription profiles and signalling pathways between VECs and the adjacent ECs from the aorta.^4^, ^6^ Additionally, there are an increasing number of studies demonstrating the phenotypic heterogeneities of VECs on the aortic surface (AS) and ventricular surface (VS) of the aortic valve. These include a differential gene transcription profile,^22^ differential production of NOS III and Cx43 proteins and the differential expression of miRNA-70 in a flow-pattern independent manner.^7^, ^8^, ^21^

VECs on the aortic valve surfaces are exposed to shear stresses during every cardiac cycle. It is certain that VECs and VICs respond to their hemodynamic environment, which can influence/determine their ability to interact with each other and to affect the remodeling of valve extracellular matrix (ECM), which is mainly composed of collagen in the fibrosa (aortic side), glycosaminoglycans (GAGs) in the middle and elastin in the ventricularis (ventricular side). The effect of laminar shear stress on the production of GAGs has been studied in the porcine aortic valve leaflets *ex vivo*.^26^ It was demonstrated that the leaflets that were subjected to laminar flow maintained the amount of GAG synthesis to a similar extent to that of fresh tissue. In contrast, the leaflet kept in a static mechanical environment had an increased production of GAGs and total proteins. The importance of interactions between VICs and VECs has also been investigated. Under both static and flow conditions, the presence of VECs increased the amount of GAGs in a scaffold containing VICs compared to scaffolds without VECs.^3^

Nonetheless, the response of side-specific VECs to the unique shear stress patterns and their effect on VIC function has not been investigated. There is, however, an interesting observation revealing the flow-independent response of VECs from the different surfaces of the valve. VECs on the aortic surface (aVECs) and those on the ventricular surface (vVECs) of the valve segments responded differently to the same patterns of shear stress, in terms of the expression of inflammatory mediators,^23^ suggesting that aVECs and vVECs have unique properties which are independent of the flow patterns.

This study aims to investigate whether there is a differential response to the flow conditions experienced by the AS and VS of the valve in terms of regulation of ECM production by the valve. With reference to levels of collagen, GAGs and elastin, the respective surfaces of porcine aortic valves were exposed to their physiological pattern of flow, as well as the reversed pattern of flow seen by the opposing side using a purpose built bioreactor.^24^

## Materials and Methods

### Porcine Aortic Valve Tissue

Porcine hearts were obtained from a slaughterhouse (Turner’s Abattoir, Farnham, Hampshire UK). The 3 leaflets of aortic valve were dissected under sterile condition with minimal contact to the surface of the valves and washed with PBS twice. Each leaflet was divided into to two equal portions through the middle of the valve in the radial direction and identical portions from the belly area of each half were dissected out for the experiments. Some samples of valve cusp were processed immediately in order to establish the ECM protein content for comparative purposes to fresh tissue (FT).

### ECM Production of Aortic Valves Under Various Mechanical Conditions

Tissue was mounted into the flow chamber dishes of a Cone-and-Plate bioreactor in order to subject the tissue to different patterns of flow.^24^ One piece of tissue, the static control (SC) was used as a reference by maintaining in the flow chamber for 48 h but not exposing it to flow conditions, while another piece of tissue, from the same leaflet, was exposed either to the oscillatory flow (−8 to +10 dyn/cm^2^ at 1 Hz) normally experienced by the aortic surface of the valve, and referred as the aortic flow (AF) or the laminar shear stress experienced by the ventricular surface of the valve (0–79 dyn/cm^2^ at 1 Hz) that is referred to as the ventricular flow (VF). It has previoulsy been shown that normal cell morphology and viability were maintained after culture in the cone-and-plate bioreactor for up to 120 h.^24^

During the static incubation and the exposure to flow conditions, tissues were maintained in DMEM containing 0.4% FBS and maintained in an incubator containing humidified air with 5% CO_2_ at 37 °C. After 48 h of static and flow incubation, the tissues were then dried at 40°C on a metal heating plate overnight, cut into small pieces and weighed before extraction and measurement of ECM components.

Collagen was extracted from the dried samples by using 10 mg/ml of pepsin (Sigma-Aldrich, UK) in 0.5 M acetic for 2 days and the content of collagen was measured using Sircol kit (Biocolor, UK). The amount of GAGs in the dried tissue was extracted and quantified by employing 20 mg/ml papain (Sigma-Aldrich, UK) and a Blyscan kit (Biocolor, UK). Elastin was solubilised from the dried tissue by 0.25 M hot oxalic acid (Sigma-Aldrich, UK) and measured by Fastin kit (Biocolor, UK).

The ECM content of fresh tissue (immediately after dissection from the heart) was also investigated to show the respective amounts of ECM prior to any intervention. The amount of ECM in the tissue that was cultured under normal shear stresses (AS/AF and VS/VF) was compared to the leaflets kept under static conditions (SC), for 48 h. Further insights in the effect of side-specific VECs and the flow patterns were studied by investigating the response of VECs to the reverse pattern of flow (AS/VF and VS/AF).

### Data and Statistic Analysis

The amount of collagen, GAGs and elastin was reported in µg per mg of (dried) tissue and represented in box-and-whiskers plot by Graphpad Prism 5. The band inside the box depicts the median. The top and bottom of the box display the third and the first quartiles, respectively, and the length of the box is the interquartile range (IQR). In these studies, the whiskers illustrate the maximum and minimum of the data set.

The groups of data were analysed statistically by a non-parametric one-way ANOVA or Kruskal–Wallis Test where appropriate. Multiple pair-wise tests were further evaluated using a Dunn’s Test. *P* values less than 0.05 were considered to be significantly different. Values for *p* and *n* numbers are given in the figure legends.

## Results

### The Effect of Shear Stresses on the Content of Collagen

**Figure 1:**
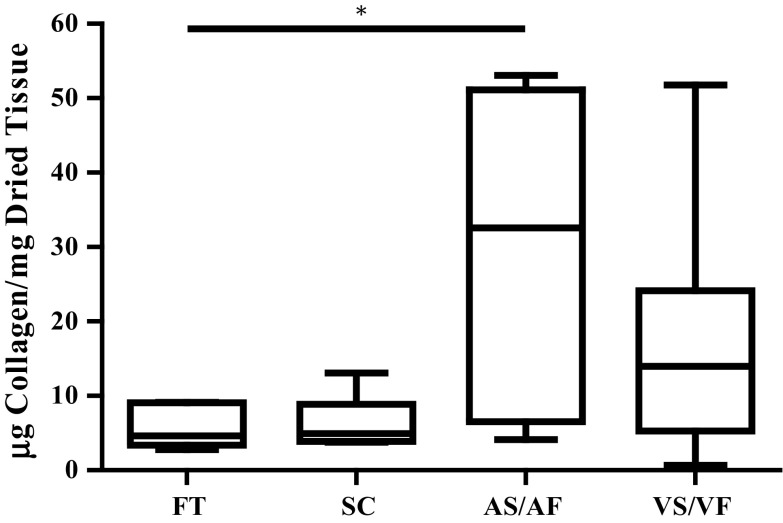
The effect of shear stresses on the amount of collagen in the aortic valves. The amount of collagen was reported in µg collagen per mg dried tissue from fresh tissue (FT), static control (SC) for 48 h and the mimicked physiological flow conditions for 48 h on the aortic side (AS/AF) and ventricular side (VS/VF). The median values were statistically analysed by Kruskal–Wallis test and pair-wise compared by Dunn’s Test. The amount of collagen in AS/AF samples was significantly higher than FT samples (*p* value < 0.05), n numbers of each group were FT = 6, CS = 6, AS/AF = 9 and VS/VF = 9.

The collagen content of leaflets that were maintained at SC for 48 h was not significantly different to that measured in FT. The median values of collagen content of FT and SC were 4.632 and 4.948 µg collagen/mg, respectively. When the AS was exposed to AF the amount of collagen significantly increased to 32.56 µg collagen/mg as compared to the FT, *p* < 0.05. In contrast, when the VS was exposed to VF there was a trend for collagen content to increase (to 13.98 µg collagen/mg), but this was not statistically different from FT or SC samples (Fig. 1).

### The Effect of Reversed Side-Specific Shear Stress on the Content of Collagen

**Figure 2:**
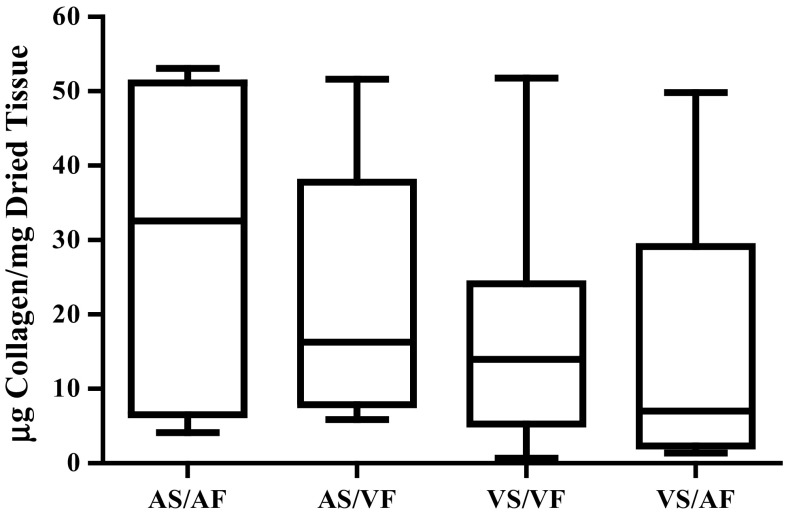
The effect of side-specific flow patterns on the amount of collagen in the aortic valves. The amount of collagen was investigated in the valves that were subjected to the normal flow (AS/AF and VS/VF) and changed flow (AS/VF and VS/AF) for 48 h. The median values were statistically analysed by Kruskal–Wallis test and pair-wise compared by Dunn’s test. There was no statistically significant difference among all groups, n numbers of each group were AS/AF = 9, AS/VF = 8, VS/VF = 9 and VS/AF = 6.

The median values of collagen content in leaflets when the AS was exposed to either the AF or VF were 32.56 and 16.28 µg collagen/mg, respectively, however this effect did not prove to be statistically significant. The median values of VS/VF and VS/AF were 13.98 and 7.039 µg collagen/mg dried tissue, respectively, which again, while changed was not significantly different (Fig. 2).

### The Effect of Shear Stresses on the Content of GAGs

**Figure 3:**
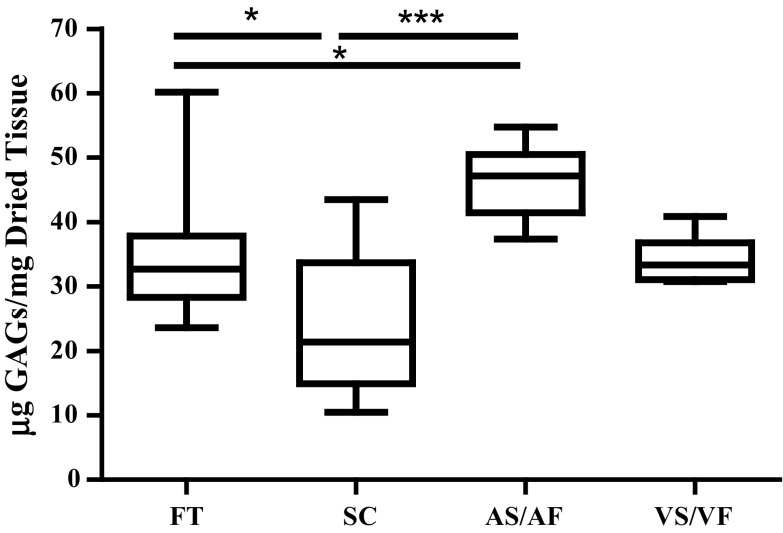
The effect of shear stresses on the amount of GAGs in the aortic valves. The amount of GAGs was reported as µg GAGs per mg dried tissue from fresh tissue (FT), static control (SC) for 48 h and the mimicked physiological flow conditions for 48 h on the aortic side (AS/AF) and ventricular side (VS/VF). The median values were statistically analysed by Kruskal–Wallis Test and pair-wise compared by Dunn’s Test. The amount of GAGs in AS/AF samples was significantly higher than FT and SC samples while the amount in SC samples was significantly reduced from FT. *P* values less than 0.05 and 0.001 were represented by * and ***, respectively. *n* numbers of each group were FT = 24, CS = 20, AS/AF = 6 and VS/VF = 6.

The amount of GAGs was significantly reduced when the leaflets were kept at the static condition for 48 h as compared to the fresh tissue (Fig. 3). The median value of FT was 32.68 µg/mg and it was reduced to 21.37 in the SC samples, *p* < 0.05. However, the amount of GAGs increased in the tissues where the AS was exposed to the AF for 48 h. The median value of 47.14 µg/mg for AS/AF tissue was significantly higher than that seen in FT and SC (*p* < 0.05 and *p* < 0.001, respectively). However, the amount of GAGs remained unchanged in the tissues when the VS was exposed to VF, with median values of 33.33 µg/mg, compared to FT or SC.

### The Effect of Reversed Side-Specific Shear Stress on the Content of GAGs

**Figure 4:**
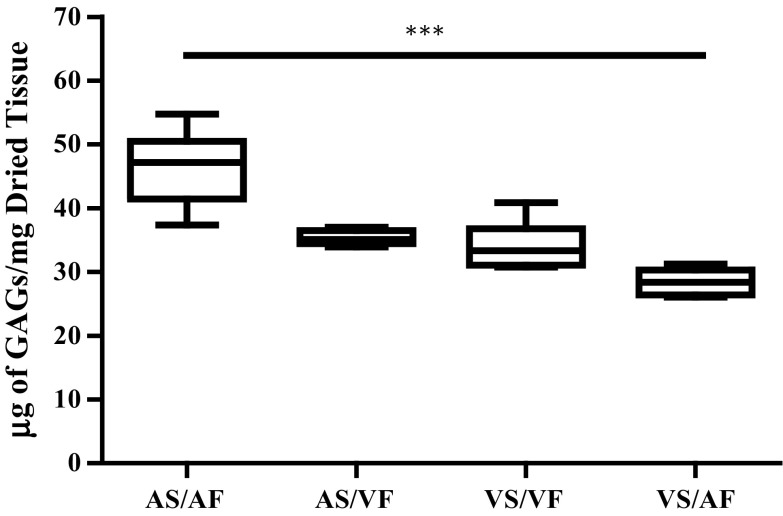
The effect of side-specific flow patterns on the amount of GAGs in the aortic valves. The amount of GAGs was investigated in valves that were subjected to the normal flow (AS/AF and VS/VF) and changed flow (AS/VF and VS/AF) for 48 h. The median values were statistically analysed by Kruskal–Wallis test and pair-wise compared by Dunn’s test. The amount of GAGs in VS/AF was significantly reduced compared to the AS/AF samples (*p* value <0.001). All samples had *n* numbers = 6.

The effect of reversed flow patterns on the amount of GAGs was assessed and shown in Fig. 4. The median value of GAG content in valves where AF was exposed to the AS for 48 h was 47.14 µg/mg, which was significantly reduced to 28.39 µg/mg when the same pattern of flow was exposed to the VS (*p* < 0.001) (Fig. 4). However, the effect of VF appears to maintain the amount of GAGs at the same level irrespective of which surface of the valve it is exposed to, with median levels of GAGs being 33.33 and 35.08 µg/mg in the VS/VF and the AS/VF groups, respectively. Moreover, the side-specific effect was not prominent. There was no significant difference observed when the AS/AF compared to AS/VF and the VS/VF compared to VS/VF, although there was a trend of decreasing amount of GAGs when the AS and VS were exposed to the reverse flow.

### The Effect of Shear Stresses on the Content of Elastin

**Figure 5:**
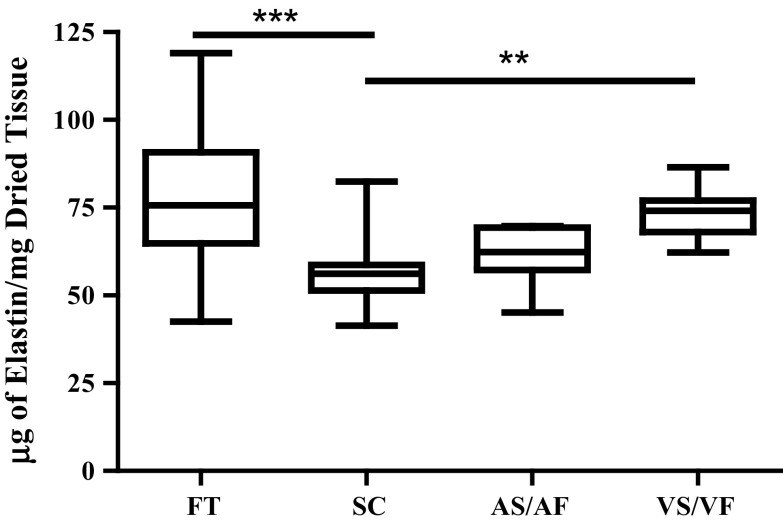
The effect of shear stresses on the amount of elastin in the aortic valves. The amount of elastin was reported as µg elastin per mg dried tissue from fresh tissue (FT), static control (SC) for 48 h and the mimicked physiological flow conditions for 48 h on the aortic side (AS/AF) and ventricular side (VS/VF). The median values were statistically analysed by Kruskal–Wallis test and pair-wise compared by Dunn’s test. The amount of elastin in VS/VF samples was significantly higher than SC (*p* value <0.01) while the amount in SC samples was significantly reduced from FT (*p* value <0.001), n numbers of each group were FT = 28, CS = 19, AS/AF = 8 and VS/VF = 9.

The amount of elastin found in the SC was significantly reduced when compared to those in the FT and in the leaflets where the VS was exposed to VF. The median value of elastin content in SC was 56.20 µg/mg, which was significantly decreased from 75.70 µg/mg in FT, *p* < 0.001 (Fig. 5). Although there was a trend of decreasing amount of elastin in AS/AF group to 62.37 µg/mg, the elastin content remained unchanged from FT, SC or the VS/VF groups. However, when the VS was exposed to VF the median elastin content rose to 74.13 µg/mg dried tissue which was significantly increased compared to the SC group, *p* < 0.01 (Fig. 5).

### The Effect Reversed Shear Stress on the Content of Elastin

**Figure 6:**
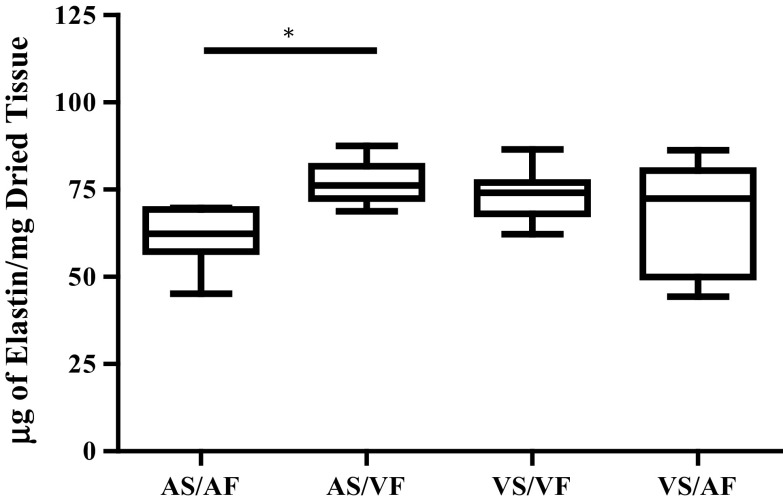
The effect of side-specific flow patterns on the amount of elastin in the aortic valves. The amount of elastin was assessed in the valves that were subjected to the normal flow (AS/AF and VS/VF) and changed flow (AS/VF and VS/AF) for 48 h. The median values were statistically analysed by Kruskal–Wallis test and pair-wise compared by Dunn’s test. The amount of elastin in AS/VF was significantly increased from the AS/AF samples (*p* value <0.05). n numbers of each samples were AS/AF = 8, AS/VF = 6, VS/VF = 9 and VS/AF = 9.

When VF was generated on the AS, it significantly increased the median value of elastin content to 76.18 µg/mg compared to 62.37 µg/mg to when the AS was exposed to AF, *p* < 0.05 (Fig. 6). The increased amount of elastin when the AS was exposed to VF was similar to when the VS was exposed to VF. However, when AS/AF was compared to VS/VF, there was no significant difference observed. Moreover when the VS was exposed to AF there was more variance in the measurement of elastin content than the VS/VF group, but the median value of 72.43 µg/mg remained unchanged from the VS/VF group.

## Discussion

This study highlights the important regulatory role that flow over the surface of the valve has on the cells within the valve. We have previously shown that the heterogeneity in the mechanical properties of VECs from each side of the valve appear to be characteristics of the cells themselves, rather than the effects of the different patterns of flow to which they are exposed.^11^ In this study we show that the responses of the valve are regulated differently by the flow patterns seen by each surface of the valve. The response of the aortic side to aortic flow patterns increases the production of collagen and GAGs, whereas the ventricular flow maintains the amount of elastin in the valve, in a side-specific manner.

The aortic valve resides in a dynamic environment and is exposed to mechanical forces during every cardiac cycle. The effects of stretch and pressure have been demonstrated to affect the production of ECM components in the aortic valve.^1^, ^2^, ^9^ In a recent study by Rathan and colleagues, porcine aortic valve cusps exposed to AF and VF showed changes in the thickness of the fibrosa in response to AF.^20^ This corresponded to histological changes in expression of collagen and elastin. They also demonstrated differential expression of miRNAs on either surface of the valve, some of which were expressed in a shear-dependant manner. The data from each study, while complementary, are difficult to compare directly due to the use of predominately qualitative data in the Rathan study and quantitative assessments of ECM production in the current study. The results of the Rathan study and those presented here both highlight the important modulatory role that the side-specific pattern of flow has on valve homeostasis.

The importance of mechanical stimulation in maintaining the ECM components in the valve is demonstrated by comparing the leaflets that were cultured under static conditions to the fresh tissue. After being kept in flow-free conditions for 48 h, the amount of GAGs and elastin were significantly reduced, which is consistent with the observations in studies on valves by other authors.^9^ In contrast, the amount of collagen remains the same in SC and FT samples, which can potentially be explained by a balance in the activity of enzymes involved in collagen synthesis and degradation.^1^, ^9^ These authors found that the production of collagenase and the activities of gelatinase (MMP-2/9) in SC for 48 h remained the same as the FT. The greater amount of collagen produced by the AS is not evident when the AS is exposed to VF. This may be due to fact that VF (on the VS) of the porcine aortic valves increases the collagenase activity of MMP-2 and 9, when compared to the control under static conditions.^16^ The expression of MMP-2 and 9 and their collagenase activities, should be investigated further to determine if these effects are due to changes in synthesis or degradation. With respect to GAGs and elastin it appears that flow serves to maintain their expression. The effect of flow on the balance between synthesis and degradation of these ECM components requires further detailed study.

Similar to the response on the collagen content, shear stress is important to maintain the amount of GAGs in the valve. The physiological conditions experienced specifically by the aortic side (AS/AF) can increase the amount of GAGs. Steady laminar shear stress maintained the amount of GAGs, as was previously demonstrated by another study.^26^ The side of the aortic valve leaflets that was exposed to ventricular flow was not identified in their studies. However, the similar results are observed in the current study, in that both VS/VF and AS/VF maintain the amount of GAGs when compared to the native valves.

Mechanical shear stress plays an important role in the regulation of the elastin content in the valves. The amount of elastin is significantly reduced when mechanical forces are absent for 48 h, but can be maintained to the same level as FT by applying VF on the VS. However, this response is not observed by the AF effect on the AS. This may be attributed to the activities of shear-sensitive elastase, cathepsin K and L, since their activities are inhibited by ventricular flow but increased by the aortic flow.^16^–^18^ This concept is further supported by the data that show the effect of VF on elastin content is not dependent on the side to which it is exposed.

There are a number of limitations to these studies. The ECM components observed are the total amount produced by VECs and VICs. Moreover, it could not exclude the effect of VECs on the side that was not exposed to the flow. For that reason, the localisation of collagen, GAGs and elastin, including flow-sensitive matrix remodelling enzymes (MMPs, TIMPs, and cathepsins) should be investigated in order to determine the response of VECs and VICs to the shear stresses. The duration of these experiments did not allow for assessment of structural and functional changes of the valve tissue used, such as changes in collagen fibre alignment/mechanical properties (stiffness), levels of collagen crosslinking in newly synthesised collagen or initiation of the calcification process. This is clearly where future work needs to be performed to accurately determine the balance between synthesis and degradation under each of the flow conditions and on each of side of the valve.

## Conclusion

This study additionally highlights the crucial role of flow patterns on the modulation of ECM components. These observations are not only important for our understanding of the complex biology and sophisticated function of heart valves, but also have important implications for those who aim to replicate their function by creating tissue engineered valves.
